# Self-Assembling Nanoparticle Hemagglutinin Influenza Vaccines Induce High Antibody Response

**DOI:** 10.3390/ijms25137259

**Published:** 2024-07-01

**Authors:** Hongying Ren, Bin Zhang, Xinwei Zhang, Tiantian Wang, Xvchen Hou, Xianyong Lan, Chuanying Pan, Jun Wu, Bo Liu

**Affiliations:** 1 Department of Microorganism Engineering, Beijing Institute of Biotechnology, Beijing 100071, China; Renhongying90@nwsuaf.edu.cn (H.R.); zhangbin@bmi.ac.cn (B.Z.); wangtiantian@bmi.ac.cn (T.W.); houxuchen@bmi.ac.cn (X.H.); 2 Key Laboratory of Animal Genetics, Breeding and Reproduction of Shaanxi Province, College of Animal Science and Technology, Northwest A&F University, Yangling, Xianyang 712100, China; xinwei.zhang@qilu-pharma.com (X.Z.); lanxianyong79@nwsuaf.edu.cn (X.L.); chuanyingpan@nwsuaf.edu.cn (C.P.)

**Keywords:** H5 subtype influenza virus, hemagglutinin, baculovirus expression system, nanoparticle vaccine

## Abstract

As a highly pathogenic avian virus, H5 influenza poses a serious threat to livestock, the poultry industry, and public health security. Hemagglutinin (HA) is both the dominant epitope and the main target of influenza-neutralizing antibodies. Here, we designed a nanoparticle hemagglutinin influenza vaccine to improve the immunogenicity of the influenza vaccine. In this study, HA5 subtype influenza virus was used as the candidate antigen and was combined with the artificially designed double-branch scaffold protein I53_dn5 A and B. A structurally correct and bioactive trimer HA5-I53_dn5B/Y98F was obtained through secretion and purification using an insect baculovirus expression system; I53_dn5A was obtained by purification using a prokaryotic expression system. HA5-I53_dn5B/Y98F and I53_dn5A self-assembled into spherical nanoparticles (HA5-I53_dn5) in vitro with a diameter of about 45 nm. Immunization and serum test results showed that both HA5-I53_dn5B/Y98F and HA5-I53_dn5 could induce HA5-specific antibodies; however, the immunogenicity of HA5-I53_dn5 was better than that of HA5-I53_dn5B/Y98F. Groups treated with HA5-I53_dn5B and HA5-I53_dn5 nanoparticles produced IgG antibody titers that were not statistically different from those of the nanoparticle-containing adjuvant group. This production of trimerized HA5-I53_dn5B and HA5-I53_dn5 nanoparticles using baculovirus expression provides a reference for the development of novel, safe, and efficient influenza vaccines.

## 1. Introduction

Avian influenza is an infectious disease in poultry caused by avian influenza virus (AIV); highly pathogenic avian influenza virus (HPAIV) can lead to high mortality rates [1]. AIV can be classified into 18 hemagglutinin (HA) and 11 neuraminidase (NA) subtypes according to the antigenicity of these surface proteins [2]. HA is an important immunogen of AIV that can stimulate the body to produce neutralizing antibodies and is an important target for vaccine development [3]. The H5 subtype of AIV is highly pathogenic to poultry and often causes severe hemorrhagic disease with a fatality rate of up to 100% [4]. An outbreak of H5N1 in 1959 in chickens in Scotland was the earliest recorded outbreak of HPAIV [5], while in 1966, there was an outbreak of H5N9 among turkey flocks in Canada [6]. The HA5 subtype of AIV continues to cause outbreaks in many places around the world. Between the beginning of 2014 to April 2015, 191 zoonotic infections of H5N1 were recorded, with 662 cases of H7N9 infections recorded since March 2013; their mortality rates were 32% and 31%, respectively (https://www.who.int/, accessed on 20 March 2024). Asia is one of the regions that has been most affected by H5N1. Two methods are at the forefront of influenza prevention strategies: culling of potential threat populations and vaccination against currently circulating strains. These tactics are both expensive and slow; in particular, culling infected populations is a costly exercise as it directly affects the bird farmers’ livelihoods and the general population by depriving them of a source of food [7]. Because of this, it is necessary to develop a vaccine to prevent and control HPAIV.

At present, many different types of influenza vaccines have been developed, including live attenuated influenza vaccines (LAIVs), inactivated vaccines, split-virion influenza vaccines, and subunit vaccines, with various other types of vaccines such as nucleic acid and viral vector vaccines in development. Quadrivalent LAIV in the United States has been shown to be effective in protecting children aged 2–17 years old [8]. In China, the first trivalent LAIV (Changchun Bcht Biotechnology Co., Changchun, China) was approved for marketing in 2020. However, LAIVs may pose a potential risk to public health, and more studies are needed to explore their safety before they can be widely used [9]. Inactivated vaccines are made by cultivating viruses in chicken embryos or mammalian cells, then inactivating them by heating or treatment with chemical agents such as formalin. Adjuvants are also added to enhance the immunogenicity of the final vaccine formulation [10]. Influenza vaccines used to prevent avian influenza in China are mainly inactivated whole influenza virus vaccines. There are six inactivated avian influenza vaccines that have been approved by the Ministry of Agriculture and Rural Affairs of the People’s Republic of China as of 2018 (http://www.moa.gov.cn/gk/, accessed on 20 March 2024), which mainly target the H5 and H7 subtypes of AIV. These six vaccines include a reassorted AIV (H5 + H7) trivalent vaccine and an inactivated (H5N2 strain rSD57 + strain rFJ56, H7N9 strain rGD76) vaccine. Split influenza vaccines destroy the viral envelope and cleave the virion, removing the lytic agent, nucleic acid, and macromolecular proteins of the virus, preserving the active antigenic components (HA and NA) along with some other viral components. Split influenza vaccines are safer than inactivated whole influenza vaccines [11,12]. Inactivated and lysed influenza vaccines for humans face problems with long production cycles, dependence on a large number of chicken embryos, and high costs. In the early stage of an outbreak, it is difficult to respond quickly enough to provide sufficient amounts of vaccine to protect animals from avian influenza. The development of recombinant protein vaccines based on conserved targets has benefitted from advances in protein crystal structure analysis. Recombinant protein vaccines help stimulate the body to produce broad-spectrum neutralizing antibodies and improve vaccine cross-protection. HA and NA are the most effective targets for neutralizing antibodies and play important roles in the development and evaluation of modern influenza vaccines. In 2013, FluBlok became the first licensed recombinant HA influenza vaccine in the United States [13]. Subsequently, clinical trials conducted during the 2014–2015 flu season showed its higher effectiveness compared with traditional inactivated vaccines [14]. Currently, research has primarily focused on the development of subunit vaccines because they are safer and easier to produce. In addition to vaccine antigens, adjuvants are sometimes added to enhance vaccine immunogenicity. Adjuvants are particularly important when developing influenza vaccines for older populations with reduced immunocompetence as well as those produced during influenza pandemics. In addition to alum, AS03, AF03, MF59, heat-resistant enterotoxin, and other vaccine adjuvants that have been incorporated into approved influenza vaccines, nanoparticles have great potential for use in influenza vaccine development because of their ability to promote slow antigen release as well as enhance vaccine immunogenicity [15].

Nanoparticles are between 1 and 1000 nm in diameter and can be effectively recognized by immune cells after entering the body [16]. Nanoparticles with a diameter of 20–200 nm have been shown to flow into lymph nodes and are readily endocytosed by resident dendritic cells, whereas larger nanoparticles (500–1000 nm) are taken up by migrating dendritic cells [17,18]. Self-assembled nanoparticles are currently a research focus, mainly based on the characteristics of some proteins that spontaneously assemble into nanoparticles. Nanoparticles have been coupled to antigens to allow for multivalent display of antigen epitopes. The size of self-assembled nanoparticles is usually between 10 and 150 nm, which is similar to the size of some viruses [19]. At present, the self-assembled proteins that are the most studied are mainly naturally occurring protein scaffolds, including serum albumin, silk protein, and ferritin. Natural components are generally chosen as vaccine carriers, though this has been limited by their restricted available number and fixed structural and chemical properties [20]. Engineering of de novo protein assemblies has become a growing field, allowing vaccine scaffolds to be more powerful and adaptable to the chosen epitopes.

The two-component nanoparticle protein scaffold I53_dn5 is a fully designed regular icosahedron with a diameter of about 24 nm. It has two components and is composed of 20 trimers of I53_dn5B and 12 pentamers of I53_dn5A for a total of 120 subunits [21]. The two components can each be expressed and purified separately, and in vitro, self-assembly can be achieved by non-covalent binding within a few minutes in an assembly buffer [22]. Studies have shown that the stable natural trimer structure of HA plays an important role in improving its immunogenicity [23]. The trimerization of HA is mainly maintained by hydrophobic interactions in the transmembrane region. However, full-length HA is a transmembrane protein, and its extraction and purification are complicated. If the transmembrane region is removed, the trimer structure will be changed [24]. The most effective way to solve this problem is to use protein recombination to replace the transmembrane domain with a non-transmembrane trimer domain. In this way, trimerized HA that resembles the natural structure can be obtained, but it can also be expressed and secreted for convenient extraction and purification. I53_dn5B is an artificially designed trimer domain that does not contain a transmembrane region and has a small molecular weight of only 14.27 kDa, making it easy to express. In 2021, a research team successfully demonstrated the HA trimers of four seasonal influenza viruses in an ordered array using the I53_dn5 scaffold, demonstrating that assembly of the two components in vitro could precisely control the display of multiple different HAs at a theoretical rate [25]. Animal experiments have shown that the nanoparticle vaccine can trigger a broader neutralizing antibody response than commercial quadrivalent influenza vaccines and can stimulate the body to produce stem-directed antibodies, effectively protecting animals from the same or different subtypes of influenza viruses. This provides new insights into the development of universal vaccines [25].

In this study, the insect baculovirus expression system was used to prepare recombinant HA5-I53_dn5B, and in vitro assembly was used with I53_dn5A prepared in a prokaryotic expression system to generate a nanoparticle vaccine. The safety and immunogenicity of the nanoparticle vaccine HA5-I53_dn5 were preliminarily evaluated by animal immunization tests. These results, based on the artificially designed I53_dn5 two-component nanoparticle scaffold protein, provide a reference for the development of novel and efficient H5 subtype influenza vaccines.

## 2. Results

### 2.1. Construction of Vectors and Expression of HA5-I53_dn5B/WT and HA5-I53_dn5B/Y98F

**Figure 1:**
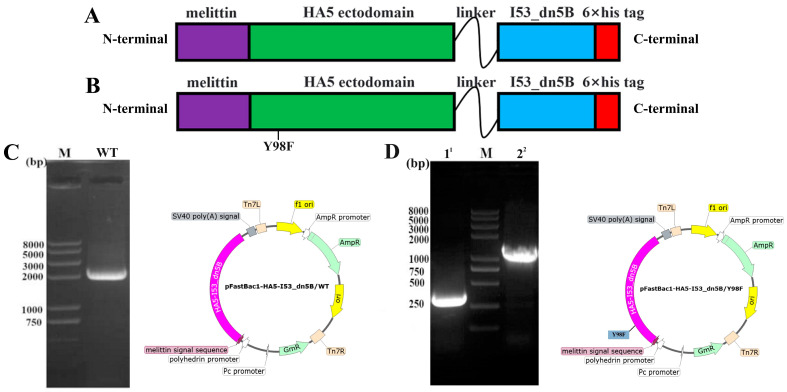
Vector construction of pFastBac1-HA5-I53_dn5B/WT and pFastBac1-HA5-I53_dn5B/Y98F. (**A**) HA5-I53_dn5B/WT expression framework diagram. (**B**) HA5-I53_dn5B/Y98F expression framework diagram. (**C**) HA5-I53_dn5B/WT fragment agarose gel electrophoresis map and pFastBac1-HA5-I53_dn5B/WT plasmid map. (**D**) HA5-I53_dn5B/Y98F fragment agarose gel electrophoresis map and pFastBac1-HA5-I53_dn5B/Y98F plasmid map. ^1^ Point mutations were introduced by PCR, and the Y98F mutation was located in lane 1. ^2^ Amplification of the remaining fragments after the introduction of Y98F mutations by primers. M stands for marker.

**Figure 2:**
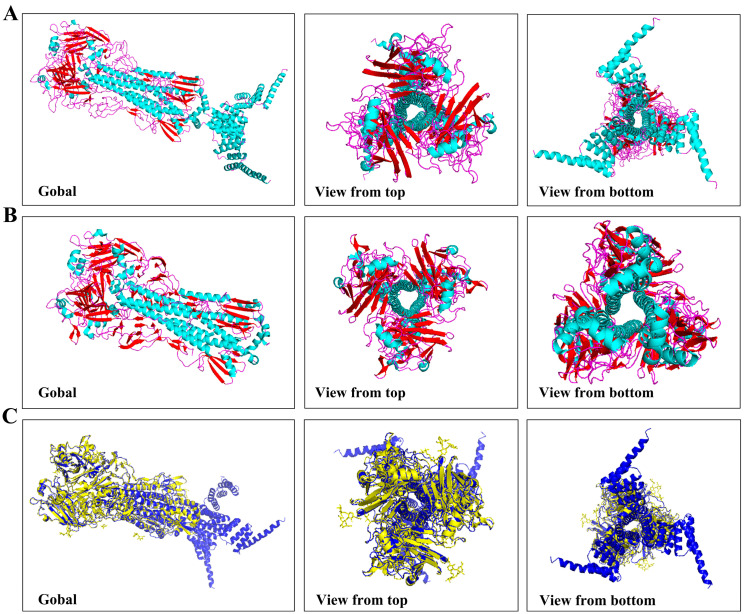
Protein 3D structure prediction. (**A**) HA5-I53_dn5B 3D structure. (**B**) HA5 3D structure. Note: Blue represents helix; red represents sheet; purple represents loop. (**C**) HA5-I53_dn5B and HA5 3D structure alignment. Note: Blue represents HA5-I53_dn5B; yellow represents HA5.

**Figure 3:**
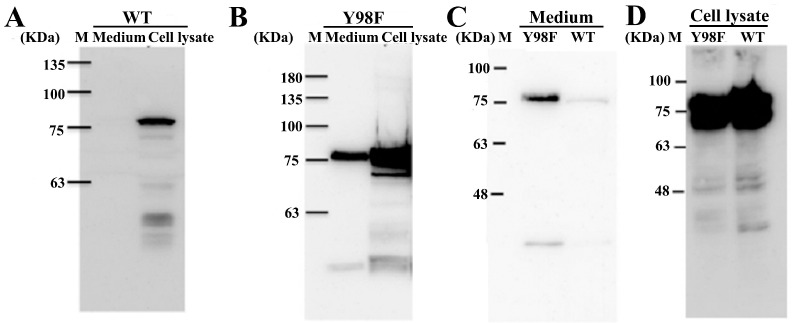
Expression of HA5-I53_dn5B/WT and HA5-I53_dn5B/Y98F. (**A**) rBac-HA5-I53_dn5B/WT protein expression assay in medium and cell lysate (anti-HA5). (**B**) rBac-HA5-I53_dn5B/Y98F protein expression assay in medium and cell lysate (anti-HA5). (**C**) Comparison of the expression of HA5-I53_dn5B/WT and HA5-I53_dn5B/Y98F in medium (anti-HA5). (**D**) Comparison of the expression of HA5-I53_dn5B/WT and HA5-I53_dn5B/Y98F in cell lysate (anti-HA5). Note: M stands for marker.

The HA5-I53_dn5B/WT fragment was successfully amplified, and the results of electrophoresis were consistent with the theoretical value of 1989 bp (Figure 1C). The HA5-I53_dn5B/Y98F mutation was successfully introduced by PCR (Figure 1D). The AlphaFold model showed that the structure of HA5-I53_dn5B was similar to that of native HA5 (Figure 2A,B). Using structural prediction and alignment, HA5 and HA5-I53_dn5B were found to remain highly structurally similar (RMSD = 0.809), which did not disrupt the trimeric structure (Figure 2C). The trimerization of HA5-I53_dn5B was maintained by the I53_dn5B domain. WB of the culture medium and cell lysates showed that HA5-I53_dn5B/WT was only present in the cells and not in the culture medium (Figure 3A), while HA5-I53_dn5B/Y98F was detected in both culture medium and cell lysates (Figure 3B). To verify this, both P1-rBACs were repackaged and then inoculated into SF9 cells of the same volume and density at the same MOI. After 72 h, the protein expression in the culture medium and cell lysate was detected again. The results of WB showed that both HA5-I53_dn5B/WT and HA5-I53_dn5B/Y98F accumulated a large amount of protein in the cells (Figure 3C,D), while the secretion of HA5-I53_dn5B/Y98F was higher than that of HA5-I53_dn5B/WT. For the remaining experiments, only HA5-I53_dn5B/Y98F, called HA5-I53_dn5B below, was secreted and purified in order to ensure protein homogeneity and ease of purification.

### 2.2. Expression, Purification, and Verification of HA5-I53_dn5B

**Figure 4:**
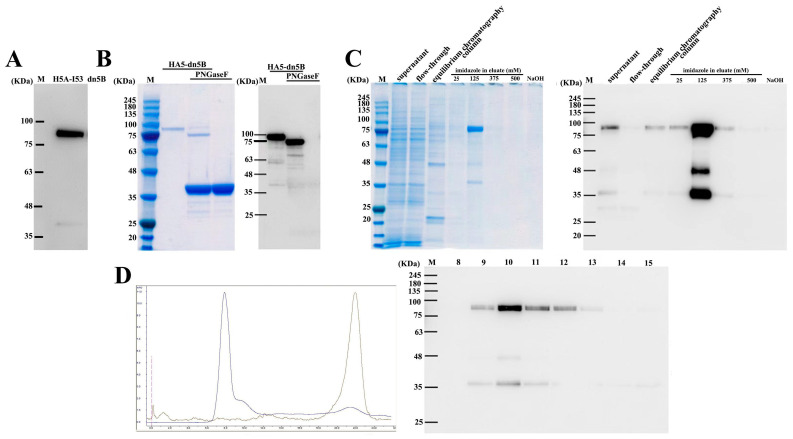
Expression, purification, and identification of HA5-I53_dn5B. (**A**) Detection of target protein HA5-I53_dn5B in SF9 cell culture supernatant. (**B**) The WB result of HA5-I53_dn5B PNGaseF enzyme digestion. (**C**) The SDS-PAGE and WB results of SF9 cell culture supernatant purified by nickel affinity chromatography. (**D**) Superdex200 increased 10/300GL gel filtration purification HA5-I53_dn5B chromatography and the WB of purified samples. Note: In Figure 4D blue curve stands for the UV curve, the yellow curve stands for the conductance curve.

PNGaseF digestion showed that HA5-I53_dn5B was successfully expressed in SF9 cells (Figure 4A), and its molecular weight was correct (Figure 4B). In initial nickel affinity chromatography, HA5-I53_dn5B was eluted mainly at 125 mM imidazole (Figure 4C). Further purification by size exclusion chromatography showed that the retention volume was about 12 mL (Figure 4D), which was consistent with the theoretical molecular weight of 225 kDa.

### 2.3. Expression, Purification, and Verification of I53_dn5A

**Figure 5:**
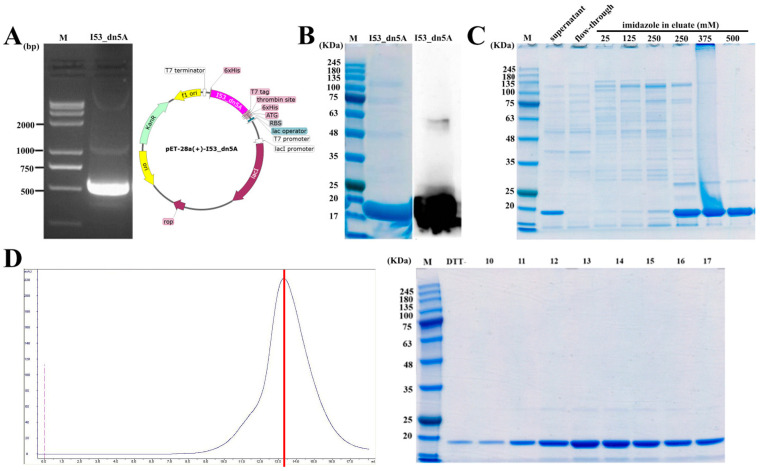
Expression and purification of I53_dn5A. (**A**) I53_dn5A fragments agarose gel electrophoresis map and plasmid map of pET28a(+)-I53_dn5A. (**B**) SDS-PAGE and WB verification of I53_dn5A expression (anti-his tag). (**C**) BL21(DE3) lysed cell supernatant purified by nickel affinity chromatography. (**D**) Superdex200 increased 10/300GL gel filtration purified I53_dn5A chromatogram and SDS-PAGE. Note: DTT stands for Dithiothreitol; In Figure 5D blue curve stands for the UV curve.

The I53_dn5A gene fragment was amplified by PCR from the synthesized pET28a(+)-I53_dn5A plasmid, and the band position was the same as the theoretical molecular weight of 471 bp (Figure 5A). After overnight induction of bacteria with IPTG, I53_dn5A was obtained from the lysed cells. Using nickel affinity chromatography, the target protein (17 kDa) was obtained at 500 mM imidazole (Figure 5B,C). After size exclusion chromatography to remove impurities, the retention volume of the target protein was about 15 mL, which was consistent with the pentamer theoretical molecular weight of I53_dn5A (85 kDa) (Figure 5D).

### 2.4. Assembly of HA5-I53_dn5 Nanoparticles In Vitro

**Figure 6:**
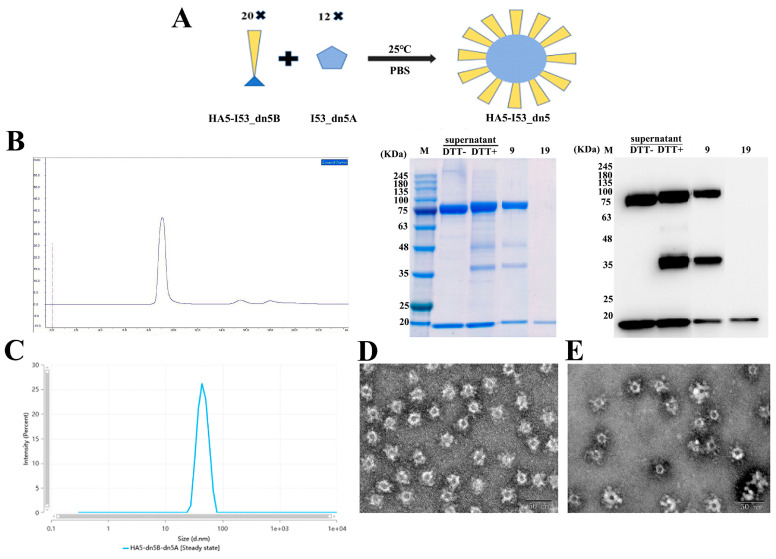
Assembly of HA5-I53_dn5 nanoparticles in vitro. (**A**) Schematic assembly of HA5-I53_dn5 nanoparticles. (**B**) Size exclusion chromatography, SDS-PAGE, and WB of size exclusion chromatography. (**C**) The particle size of HA5-I53 dn5 determined by DLS. (**D**) The dn5 morphology of HA5-I53 was observed by TEM with bar = 50 nm. (**E**) The dn5 morphology of HA5-I53 was observed by TEM with bar = 50 nm after 30 days of storage. Note: DTT stands for Dithiothreitol. In Figure 6B blue curve stands for the UV curve.

When purified HA5-I53_dn5B and I53_dn5A were mixed and repurified, the retention volume was 9 mL, indicating the formation of nanoparticles. Unassembled components appeared at 19 mL (Figure 6B). DLS results showed that the diameter of the nanoparticles was about 40 nm, with good uniformity (Figure 6C). TEM indicated that the nanoparticles were spherical, with a diameter of about 40 nm, and HA5 was seen on the surface of the nanoparticles as spikes (Figure 6D). After storing HA5-I53_dn5 at 4 °C for 30 days, HA5 was still displayed on the surface of the nanoparticles in the form of spikes, and no obvious cleavage was observed (Figure 6E).

### 2.5. Evaluation of the Immunogenicity in the Mouse Model

In the immunization experiment, the weight change trends of mice in all immunized groups were the same. Body weights generally increased over time, and there were no adverse reactions, which preliminarily suggested that the vaccine has a good safety profile. The reason for the discrepancy in the actual weights is speculated to be because the mice were not stationary during weighing; their activity likely introduced some error into the measured weights. The total IgG antibody titer in the HA5-I53_dn5 + Al(OH)_3_ group 3 weeks after the first immunization was significantly higher than that in the HA5-I53_dn5B + Al(OH)_3_ group (*p* < 0.05). The total IgG titer of the serum in all groups was increased 3 weeks after the second immunization. There was no significant difference between the HA5-I53_dn5B + Al(OH)_3_ group and the HA5-I53_dn5 high-dose group (*p* > 0.05), where antibody titers were significantly lower than that in the HA5-I53_dn5 + Al(OH)_3_ group (*p* < 0.05). High titers of IgG1 and IgG2a were induced by 1 µg HA5-I53_dn5B, 5 µg HA5-I53_dn5B, 1 µg HA5-I53_dn5, and 5 µg HA5-I53_dn5. The IgG2a titer was significantly higher in the 5 µg HA5-I53_dn5 group than in the other groups (*p* < 0.05).

## 3. Discussion

Self-assembling proteins can be used as nanoparticles mediating multi-copy antigen display. This multivalent molecular setting favors the fruitful network of stimulatory interactions, as opposed to the weaker effect of monovalent binding afforded by single soluble recombinant antigens. The high avidity for the nanoparticle provided by the multivalent interaction constitutes a critical step in the induction of a potent immune reaction. Compared with traditional vaccines, nanoparticle vaccines have stable structure, high targeting, and strong immunogenicity; can accumulate in lymph nodes; have advantages in antigen assembly and antigen delivery, as multiple immune factors can be combined in an orderly manner; and also have unique pathogen mimicry properties [26]. mRNA vaccines deserve particular attention, as their use in humans began recently with the COVID-19 pandemic. mRNA vaccines are administered in the form of nanoparticles where the RNA material is protected by an outer lipid envelope that will eventually facilitate cellular entry. From the point of view of immunogenicity, mRNA vaccines can produce both humoral and cellular immunity in vaccinated individuals and have a strong immune response [27,28]. This is in part similar to the role of nanoparticle vaccines. However, a known disadvantage of these vaccines is that mRNA is less stable and prone to degradation. In short, self-assembled nanoparticle vaccines are safe, can provoke effective immune response, and are economical and stable. However, the self-assembled nanoparticles vaccine has a requirement for the expression system; some need adjuvant and limitation of antigen space and conformation. mRNA vaccines without virus replication can stir up effective immune response and is cost-effective and stable. However, the mRNA vaccine does not allow for genetic errors and is fragile and has delivery limitations, which limits its application [29].

In this study, the extracellular domain of HA5 was fused with I53_dn5B, and secreted expression was successfully achieved using the baculovirus expression system. The purified HA5-I53_dn5B was verified and could maintain its trimeric structure and biological function. By comparing reducing and non-reducing SDS-PAGE and WB results, part of HA5-I53_dn5B was seen to decompose into an HA1 subunit of ~48 kDa, while the rest was ~35 kDa. Similar results were also observed previously when full-length HA5 was expressed using the insect baculovirus expression system [30]. HA is a trimeric surface glycoprotein involved in viral invasion; each of its monomers consists of two subunits (HA1 and HA2) that are linked by disulfide bonds. HA1 is made up of a spherical head, while the other part is a stem structure composed of part of HA1 and all of HA2 [31,32]. β-mercaptoethanol cleaves intermolecular disulfide bonds, which resulted in HA bands around 35 and 48 kDa. This showed that part of the HA5-I53_dn5B/Y98F purified using this method was in its mature conformation. The HA1 and HA2 subunits were then cleaved by protease to form disulfide bonds. Some proteases in the insect cells may play a role in this assembly process. Studies have found that after the mutation of Y98F in HA, the hydroxyl group in that position disappears, resulting in a failure to form hydrogen bonds with the sialic acid (SA) receptor and eliminating this interaction. This mutation has been validated in H1, H3, and H5 and has been shown to not affect HA folding and integrity [33,34]. In this study, both the Y98F mutant and wild-type HA5-I53_dn5B were constructed, but it was found that only a small amount of HA5-I53_dn5B/WT was secreted; Y98F mutation effectively promoted its secretion, though the reasons behind this need to be further clarified. Y98F mutation has been introduced before to facilitate protein expression and purification of HA [35].

Both HA5-I53_dn5B/Y98F and HA5-I53_dn5 stimulated the production of high titer HA5-specific antibodies in mice without adjuvants, resulting in a strong humoral immune response. It is generally believed that recombinant proteins have poor immunogenicity and can only cause low-intensity immune responses [36]. However, the present study found that the two subunit vaccines could induce strong humoral immune responses, which may be related to their higher antigen density. The most commonly used and stable Al(OH)_3_ adjuvant was also tested; it further enhanced the immunogenicity of the two vaccines, reflecting the important role of adjuvants in immune activation. HA5-I53_dn5 produced higher antibody titers than the same dose of HA5-I53_dn5B/Y98F; there was no significant difference between the antibody titers of the 5 µg HA5-I53_dn5B/Y98F ± Al(OH)_3_ groups. This might be because of the high density of HA5 on the surface of HA5-I53_dn5, which crosslinks to activate BCR, resulting in higher B cell proliferation and antibody production [37]. Overall, this showed that the nanoparticle had significant advantages in immune activation, effectively replacing the adjuvant; nanoparticles also reduce the number of antigens, avoiding side effects and reducing costs.

Antibody typing results showed that antibody titers of IgG1 and IgG2a in all vaccine groups were significantly higher than those in the control group. IgG1 and IgG2a are Th2 and Th1 cell markers, respectively, indicating that both vaccines could induce Th1 and Th2 immune responses. Higher titers of IgG2a were observed in the 5 µg HA5-I53_dn5 group, suggesting that nanoparticle elicits a stronger Th1-type immune response, potentially more effectively clearing the virus. In summary, HA5-I53_dn5 is a spherical nanoparticle with a particle size of 45 nm and good homogeneity. Studies have shown that nanoparticle vaccines typically enter the body through the intramuscular route for lymph node targeting, where they are enucleated and presented by APCs with high efficiency [38]. This activates humoral and cellular immunity, effectively solving the problem of the low immunogenicity of recombinant protein vaccines.

## 4. Materials and Methods

### 4.1. Cell Cultures

Spodoptera frugiperda cells (SF9) (Gibco, Waltham, MA, USA) were cultured in SF900 II SFM (Gibco, USA) medium in a CO_2_-free, non-humidified 27 °C incubator at 125 rpm.

### 4.2. Construction of Recombinant Shuttle Plasmid Bacmid-HA5-I53_dn5B

The amino acid sequence of H5N6 (A/cat/Sichuan/SC18/2014; GenBank: AIU46599.1) was obtained from NCBI (www.ncbi.nlm.nih.gov). The I53_dn5B amino acid sequence was obtained from a previous study [25]. HA5 and I53_dn5B amino acid sequences were synthesized by Bio Inc (Sangon, Beijing, China). The extracellular domain of HA5 was fused with the I53_dn5B domain using a flexible linker GGSV; the resulting vector was named pFastBac1-HA5-I53_dn5B/WT (Figure 1A). As the Y98F (HA3 numbering) mutation in HA can eliminate receptor binding activity and improve its immunogenicity [39], this mutation was also introduced in HA5-I53_dn5B. The location of Y98F mutation was determined according to the conservation of H3 sequence, and point mutation primers were designed (Table 1). The Y98F mutation was introduced by polymerase chain reaction (PCR), and the target fragment was amplified in two fragments. The mutant vector was called pFastBac1-HA5-I53_dn5B/Y98F (Figure 1B).

pFastBac1-HA5-I53_dn5B/WT and pFastBac1-HA5-I53_dn5B/Y98F were transferred into DH10BAC cells (TransGen, Beijing, China) [40]. Blue-white spot screening and polymerase chain reaction were used to identify positive clones [40]. Plasmids were extracted using an endotoxin-free plasmid extraction kit (Tiangen, Beijing, China), and the extracted shuttle vectors were named Bacmid-HA5-I53_dn5B/WT and Bacmid-HA5-I53_dn5B/Y98F. AlphaFold2 (London, UK) was used to predict the 3D structure of HA5-I53_dn5B.

### 4.3. Culture and Purification of Recombinant Baculovirus

Bacmid-HA5-I53_dn5B was transfected into SF9 cells using Lipofectin 3000 (Invitrogen, Waltham, MA, USA), and after 5 days of culture, the supernatant was extracted by centrifugation, resulting in a first-generation recombinant baculovirus (rBac-HA5-I53_dn5B). The first-generation virus was used to infect SF9 (multiplicity of infection (MOI) = 0.1), preparing a second-generation virus with high titer. The prepared virus was stored at 4 °C away from light and was used within one month.

### 4.4. Expression and Purification of HA5-I53_dn5B

When SF9 cell density reached 2 × 10^6^ cells/mL, the two generations of rBac-HA5-I53_dn5B were added (MOI = 5). The supernatant was removed for protein purification after continuous culture for 72 h.

The target proteins were obtained using nickel affinity chromatography. The chromatographic column was washed twice with a solution of 20 mM Tris (pH 7.5), 500 mM NaCl, and 5 mM imidazole before elution of His-tagged protein using a solution of 20 mM Tris-HCL (pH 7.5), 500 mM NaCl, and 500 mM imidazole. Eluates were concentrated and applied to a Superdex 200 Increase 10/300 GL column pre-equilibrated with PBS for preparative size exclusion chromatography. Peaks corresponding to trimeric species were identified based on elution volume and sodium dodecyl sulfate-polyacrylamide gel electrophoresis (SDS-PAGE) of elution fractions. To verify whether the prepared HA5-I53_dn5B had N-glycosylation modification, the protein was digested with peptide-N-glycosidase F (PNGaseF) (Yeasen, Shanghai, China).

### 4.5. Production of I53_dn5A Antigens

The synthesized pET28a(+) vector plasmid containing the dn5A sequence [25] was transformed into *Escherichia coli* expression strain BL21(DE3) (Tiangen, China) using heat shock. The transformed bacteria were inoculated into 3 mL Luria-Bertani broth (30 µg/mL kanamycin) and cultured in a shaking incubator at 210 rpm for 8 h at 37 °C. The cultured bacterial solution was transferred to 250 mL of the same medium at a 3% inoculation, and culturing was continued in a shaking incubator at 37 °C until the OD_600_ reached 0.6. Isopropyl β-D-thiogalactoside (IPTG) was added at a final concentration of 0.5 mmol/L, and the temperature was lowered to 18 °C overnight to induce protein expression. The induced culture was then centrifuged at 12,000× *g* for 30 min, the supernatant was discarded, and the equilibrium solution (20 mM Tris-HCL pH 7.5 and 500 mM NaCl) was added to resuspend the bacteria. The volume of the equilibrium solution (mL) was about 10 times the centrifugated weight of the bacteria (g). Then the cells were lysed using ultrasound for 30 min in an ice bath. The lysed cells were centrifuged at 4 °C at 12,000× *g* for 30 min, and the precipitate was removed; the supernatant was used for subsequent protein purification. Nickel affinity chromatography was performed using a Ni Sepharose 6 Fast Flow column, and the purest components containing the target protein (as determined by SDS-PAGE and western blot (WB)) were concentrated to 1 mL using an ultrafiltration tube with a cut-off of 10 kDa. Finally, a Superdex 200 Increase 10/300GL column was used for size exclusion chromatography to further purify I53_dn5A.

### 4.6. Assembly of Nanoparticles In Vitro

Both HA5-I53_dn5B/Y98F and I53_dn5A proteins were diluted to 500 µg/mL in PBS, and were then mixed at a ratio of 1:1 and incubated in a constant temperature shaker at 125 rpm for 1 h at 25 °C. Nanoparticles and unassembled components were separated using Superose 6 Increase 10/300 GL chromatographic columns; the mobile phase was PBS, and the flow rate was 0.5 mL/min. Components with different retention times were collected according to the position of the ultraviolet absorption peak and tested by SDS-PAGE and WB. The assembled nanoparticle was named HA5-I53_dn5 and was stored at 4 °C for 30 days. Dynamic light scattering (DLS) and transmission electron microscopy (TEM) were used to detect the structural integrity of the HA5-I53_dn5 protein.

### 4.7. Experiments with Mice

A booster immunization strategy at a three-week interval was used to evaluate and compare the immune efficacy of trimer and nanoparticle HA5 vaccine. Seventy SPF grade 6-week-old female BALB/c mice (Beijing Vital River Laboratory Animal Technology Co., Ltd., Beijing, China) were fed in the barrier environment of the Experimental Animal Center of the Beijing Institute of Biotechnology (Beijing, China) for a week to fully adapt to the environment. All animal experiments were conducted in accordance with the guidelines of the Animal Care and Use Committee of the Animal Center of the Beijing Institute of Biotechnology (Experimental Animal Welfare Ethics approval number: IACUC-DWZX-2020-039).

**Figure 7:**
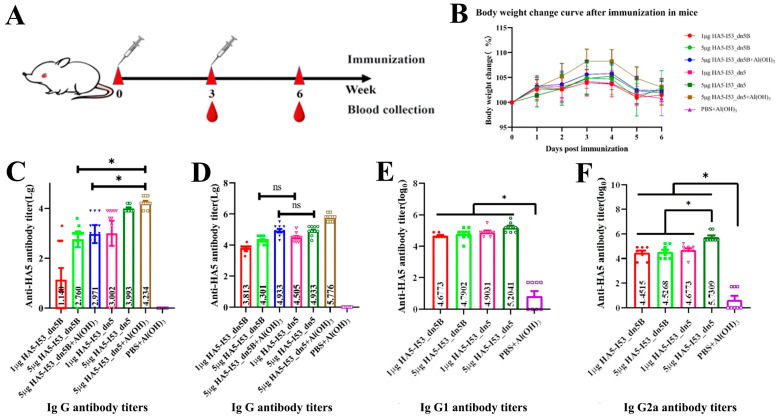
Evaluation of mouse immunity. (**A**) Flow chart of mouse immunization. (**B**) Body weight change curve post first immunization. (**C**) IgG antibody titers in serum 3 weeks after the first immunization. (**D**) IgG antibody titers in serum 3 weeks after the second immunization. (**E**) IgG1 antibody titers in serum 3 weeks after the second immunization. (**F**) IgG2a antibody titers in serum 3 weeks after the second immunization. Note: * *p* < 0.05, ns stands for no differences.

A total of six experimental groups and one control group were designed (Table 2). To compare the effect of adjuvant and nanoparticles on vaccine immunogenicity, a vaccine group with Al(OH)_3_ adjuvant (Croda, Denmark) was also designed. The mice were randomly divided with ten mice per group. This study adopted an immune-boost strategy (Figure 7A). The mice were given intramuscular injections on days 0 and 21. After the first immunization, weights were recorded each day. Blood was collected 21 days after the first immunization and 21 days after enhanced immunization. Serum was isolated from collected blood from each mouse and stored at −80 °C for subsequent use.

### 4.8. Detection of HA5-Specific Total IgG, IgG1, and IgG2a Antibody Titers

Serum titers of HA5-specific total IgG, IgG1, and IgG2a were detected by indirect enzyme-linked immunosorbent assay (ELISA). The HA5 protein of H5N6 (A/Sichuan/26221/2014, SinoBiological, Beijing, China) was diluted to 0.5 μg/mL with a coated solution, and 100 μL was added to each well of the ELISA plate and incubated overnight at 4 °C. The liquid from each well was then discarded, and the plate was washed three times with phosphate-buffered solution (PBST) and patted dry. Skim milk (200 μL of 5%) was added to each well, and the plate was incubated at 37 °C for 1 h. The milk was discarded, and 100 μL of sealer was added to each well in columns 2 to 12. The serum from each group was diluted into 200 μL of sealing solution at a ratio of 1:5000 and added to each hole in column 1. The first column of serum was mixed, and then 100 μL was taken from these wells and added to those of the second column and then carried out successively until the 11th column. Column 12 had blank serum added as a control. After incubating at 37 °C for 1 h, the serum was discarded, and the ELISA plates were washed with PBST five times. Goat Anti-Mouse IgG H&L (HRP), Goat Anti-Mouse IgG1 (HRP), and Goat Anti-Mouse IgG2a heavy cHIn (HRP) (Abcam, Cambridge, UK) were respectively used in sealing solution (1:5000) before 100 μL of diluted secondary antibody was added to each well. After incubation at 37 °C for 1 h, the secondary antibody was discarded, and the ELISA plates were washed with PBST five times. Finally, 100 μL of 3,3′,5,5′-tetramethylbenzidine (Beyotime, Shanghai, China) was added into each well for 5 min, and then 50 μL 2M H_2_SO_4_ was added into each well to terminate the chromogenic reaction, and absorbance at 450 nm was measured. The ELISA end-point titer was defined as the highest serum dilution that exceeded the background value at 450 nm by a factor of 2.1 and was converted to log10 for analysis.

### 4.9. Statistical Analysis

All results are shown as mean ± standard error. Statistical analysis and plotting were performed using GraphPad Prism 8.0 (GraphPad, La Jolla, CA, USA). When variance was homogeneous among the data of each group, a one-way analysis of variance was used for analysis, and Dunnett’s method was used for multiple comparison. The non-parametric Kruskal–Wallis test was used for analysis when variance was inconsistent among the data in each group. *p* < 0.05 indicates a significant difference.

## 5. Conclusions

Recombinant HA5-I53_dn5B and I53_dn5A were expressed by insect baculovirus and prokaryotic expression systems, respectively. The particles self-assembled into HA5-I53_dn5 nanoparticles in vitro. It was preliminarily demonstrated that trimerized HA5-I53_dn5B nanoparticle vaccines have good immunogenicity and can induce a humoral immune response, which lays a foundation for the development of novel and efficient H5 subtype influenza vaccines.

## Figures and Tables

**Table 1:** Primer design for point mutation.

Name	Forward Primer (5′-3′)	Reverse Primer (5′-3′)
HA5-I53_dn5B/Y98F-1	AAGGCCTACGTCGACGAGCT	AGTCGTTCAGGTTACCAGGGA AGCACAGGTCGTTAGCAGGG
HA5-I53_dn5B/Y98F-2	CCCTGGTAACCTGAACGACT	CTTGGTACCGCATGCCTCGA

Underscore indicates the mutated base.

**Table 2:** Information on mouse immunization groups.

Serial Number	Grouping	HA (µg)	Al(OH)_3_ 10 mg/mL (µL)	Immune Volume (µL)	Numbers
1	HA5-dn5 Low	1	0	100	10
2	HA5-dn5 High	5	0	100	10
3	HA5-dn5 High + Adjuvant	5	10	100	10
4	HA5-dn5B Low	1	0	100	10
5	HA5-dn5B High	5	0	100	10
6	HA5-dn5B High + Adjuvant	5	10	100	10
7	PBS	0	10	100	5

Low stands for low-dose antigen. High stands for high-dose antigen.

## Data Availability

Data will be made available on request.
